# Clinical Decision Support and New Regulatory Frameworks for Medical Devices: Are We There Yet? A Response to the Recent Commentaries

**DOI:** 10.34172/ijhpm.2023.8388

**Published:** 2024-01-07

**Authors:** Katoo M. Muylle, Audrey Van Scharen, Veronique Shiwa, Pieter Cornu

**Affiliations:** ^1^Research Group Clinical Pharmacology and Clinical Pharmacy (KFAR), Centre for Pharmaceutical Research (CePhar), Vrije Universiteit Brussel (VUB), Brussels, Belgium; ^2^Medical Ethics Committee, Universitair Ziekenhuis Brussel, Brussels, Belgium; ^3^Gerontological Sciences (GERO), Vrije Universiteit Brussel (VUB), Brussels, Belgium

 In our viewpoint paper we outlined the United States’ and the European Union’s (EU’s) regulatory response on the rapidly changing digital health landscape with specific focus on the impact on clinical decision support (CDS) systems as a type of medical device software. Further challenges have been raised in subsequent commentaries.^1-3^ In this correspondence, we discuss some of the many remaining challenges, and provide further thoughts on the regulation of artificial intelligence (AI) in healthcare.

 A first challenge raised by Beckers and Van Hoydonck,^1^ is the influence of the regulation on the portfolio of medical software manufacturers. Because of the more stringent Medical Device Regulation (MDR), software manufacturers evaluate the return on investment and for some products, manufacturers have decided to leave the EU market. The discrepancy between the EU MDR and Food and Drug Administration risk classification for CDS software that informs healthcare practitioners for non-serious patient conditions still exists resulting in a much higher regulatory burden for these type of CDS systems in the EU.^1,4^ The cost and time-consuming process of MDR certification places Europe at a disadvantage against international competitors, thereby potentially hampering innovation and causing shortages of medical software. When only a limited number of functionalities of the software result in its classification as a medical device, software manufacturers evaluate whether these functionalities can be omitted.

 A second challenge raised by Maresova^3^ is the shortage of notified bodies within the EU. At the time of writing our viewpoint paper, there were only 23 notified bodies spread over 11 different countries in the EU accredited to regulate all applications for medical devices.^4^ Meanwhile, this number has increased to 42 in 18 different countries.^5^ Despite the increase, the shortage remains. Under the EU Medical Device Directive, manufacturers self-certified Class I software.^6^ As more software is classified into higher classes under the MDR, many manufacturers need a notified body for the first time.^7^ This created a significant increase in regulatory workload for notified bodies. It is especially difficult for smaller enterprises to hire a notified body because these notified bodies are at the limit of their capacity, and some have a long waiting list or do not even accept new applications.^8^ In addition, it is even more difficult for small and medium-sized enterprises (SMEs) to access notified bodies and obtain CE marking for market access if notified bodies compete and do not take into account the interests of SMEs in relation to fees, as required by the MDR.^7,9^ Because the capacity of notified bodies is still not sufficient to ensure certification of all Medical Device Directive legacy devices, the transition period for legacy medical software is extended to December 31, 2027 for high-risk medical software or December 31, 2028 for medium and low risk medical software.^8^

 A third question is whether the practical implementation of the regulations has become clearer. Medical device software manufacturers struggle to find support to fulfill all the necessary steps for a complete submission file. An important one being the clinical investigations, proving its safety and performance. To this end companies often seek cooperation with academic centers. The company proposes to put the uncertified device at the disposition of researchers, stating the researcher can use the device in their research for free. In return the company wants to use the results of the clinical investigation. What enthusiastic researchers often do not realize is that by accepting this offer, they must fulfil the heavy regulatory requirements of clinical investigations with a non-certified device.

 Keeping track of all the regulatory requirements in research with software medical devices has become a regulatory minefield in the EU. The framework consists of a mix of legal norms (eg, legislation on scientific experiments, medical devices, data protection, health technology assessment, and AI), ethics principles and deontological rules (or so-called scientific integrity rules/good clinical practice rules) in practicing science and medicine. The intended harmonization of the MDR failed when it came to clinical investigations, as every member state could further develop their own regulatory framework for seeking ethics approval. In Belgium, the same heavy regulatory framework applies for academic clinical research not aiming to get conformity certification. The suggestion has been made to create an academic discipline of “medical device science.”^10^

 A last challenge is the rapidly changing landscape of AI in healthcare. McKee and Wouters^2^ described in detail challenges when regulating AI in healthcare. Recently, the European Council and Parliament reached a provisional agreement on the content of the AI Act, the first in its kind.^11^ This AI Act aims to balance innovation, transparency, and safety by introducing a risk-based approach whereby AI tools considered as having an unacceptable risk to users such as social scoring will be banned. Strict requirements on data quality and accuracy, transparency and traceability, accountability and human oversight are imposed on high-risk AI systems.^11,12^

 For medical device software using AI, the AI Act can be considered complementary to the MDR. The AI Act stipulates extra elements of focus with regards to the AI component of the software. Both regulations apply a risk-based approach. In the AI Act, medical devices are explicitly mentioned as an example of high-risk AI tools. This would mean that even though a medical device using AI is considered medium risk under the MDR, it would be considered high risk under the AI Act. The intention is that conformity assessment of Class IIa or higher medical devices under the AI Act is conducted by notified bodies already in charge of conformity assessment under the MDR.^12^ Since the AI Act imposes additional requirements compared to the MDR, the conformity procedure for AI based medical device software will be more demanding and complex compared to expert rule based medical device software with the same intended use.

 Innovation often stems from tailor-made and agile solutions developed by SMEs. To alleviate the burden on SMEs and to foster innovation, the AI Act provides some derogations. So-called regulatory sandboxes will act as controlled environments to enable SMEs to develop, train, and validate compliant AI systems. Under specific conditions and safeguards, testing of AI systems in real-world conditions will also be allowed as a measure to support innovation. Adequate and user-friendly implementation of these sandboxes will be key in securing Europe’s competitiveness in the AI-based medical device market. To conclude, the EU AI Act undoubtedly promotes trustworthy and human-centric AI-tools, but if it will foster responsible innovation in Europe remains to be seen.

## Ethical issues

 Not applicable.

## Competing interests

 Pieter Cornu and Audrey Van Scharen are members of the Medical Device Framework Board of the UZ Brussel and VUB.
